# Atherosclerotic disease is increased in recent-onset rheumatoid arthritis: a critical role for inflammation

**DOI:** 10.1186/ar2323

**Published:** 2007-11-06

**Authors:** Suad Hannawi, Brian Haluska, Thomas H Marwick, Ranjeny Thomas

**Affiliations:** 1 Centre for Immunology and Cancer Research, University of Queensland, Princess Alexandra Hospital, Brisbane, QLD 4102, Australia; 2 Department of Medicine, University of Queensland, Princess Alexandra Hospital, Brisbane, QLD 4102, Australia; 3 Diamantina Institute for Cancer, Immunology and Metabolic Medicine, University of Queensland, Princess Alexandra Hospital, Brisbane, QLD 4102, Australia

## Abstract

Rheumatoid arthritis (RA) patients have increased mortality and morbidity as a result of cardiovascular and cerebrovascular disease. What is not clear, however, is either how early accelerated atherosclerosis begins in RA or how soon risk factors must be rigorously controlled. Furthermore, given the strong relationship of vascular disease to RA mortality and of inflammation to the accelerated atherosclerosis associated with RA, it is important to evaluate indices that could serially and noninvasively quantify atherosclerotic disease in RA patients. The carotid intima-media thickness (cIMT) and plaque, measured by ultrasound, correlate closely with direct measurement of the local and systemic atherosclerotic burden. To investigate the presence of subclinical atherosclerosis in the early stages of RA, the cIMT and plaque were measured using carotid duplex scanning in 40 RA patients with disease duration < 12 months and in 40 control subjects matched for age, sex and established cardiovascular risk factors. Patients with RA had significantly higher average cIMT values and more plaque than the control group (cIMT 0.64 ± 0.13 mm versus 0.58 ± 0.09 mm, respectively; *P *= 0.03). In RA patients, the cIMT was predicted by age and C-reactive protein level at first presentation to the clinic (*R*^2 ^= 0.64). C-reactive protein was associated with age of disease onset and history of smoking. Since inflammation has been shown to predate onset of clinical RA, the accelerated atherogenic process related to inflammation may precede RA symptom onset.

## Introduction

Rheumatoid arthritis (RA) patients have significantly increased mortality and morbidity as a result of cardiovascular (CV) disease and cerebrovascular disease [1]. Laboratory, clinical and epidemiological studies suggest that immune dysregulation and systemic inflammation play important roles in the accelerated atherosclerosis of RA [2,3]. Although CV events are major consequences of CV disease, these complications develop over years, and the time course of epidemiologic and clinical studies has been reduced using B-mode ultrasound measurement of the carotid intima-media thickness (cIMT) and of carotid plaque to study early atherosclerotic changes noninvasively [4,5]. cIMT values measured by ultrasound correlate closely with direct measurement of the local and systemic atherosclerotic burden in pathology studies and with clinical CV endpoints [4,6]. Ultrasonographic assessment of common carotid atherosclerosis is a feasible, reliable, valid and cost-effective method for both population studies and clinical trials of atherosclerosis progression and regression [7].

While several studies have demonstrated subclinical atherosclerosis in established RA patients, either by increased cIMT or the presence of carotid atherosclerotic plaque [8-14], no study has examined the cIMT or plaque in early RA. This is an important issue for several reasons. How early accelerated atherosclerosis begins in RA, or how soon risk factors must be rigorously controlled, is not clear. Furthermore, given the strong relationship of CV disease to RA mortality, it is important to evaluate indices that could serially and noninvasively quantify atherosclerotic disease in RA patients. Given the clear association of inflammation with accelerated atherogenesis and CV clinical outcomes in RA, we hypothesized that the cIMT would be increased in RA patients even at an early stage of RA, related to inflammatory factors associated with RA. To investigate the presence of subclinical atherosclerosis in the early stages of RA, we measured the cIMT in RA patients with symptom duration <12 months and in a group of control individuals matched for age, sex and CV risk factors. A group of conventional CV risk factors and indices of RA inflammatory disease were analysed to determine their relationship to subclinical atherosclerosis in RA patients.

## Materials and methods

### Rheumatoid arthritis patients

Patients (*n *= 40), including 13 men and 27 women with a mean age of 53 years (range 22–78 years), were recruited at first presentation between May 2004 and December 2005 when attending scheduled appointments at our specialized early RA clinic. All patients aged 18 years or older attending the early RA clinic during the study recruitment period were invited to take part in the study. All participants met the American College of Rheumatology 1987 revised criteria for diagnosis of RA. At presentation, five patients were taking low-dose prednisone, and three patients were using nonsteroidal anti-inflammatory drugs or cyclooxygenase-2 inhibitors. Unless contraindicated, all RA patients were started on triple therapy of methotrexate, sulphasalazine and hydroxychloroquine after RA diagnosis was confirmed, and the doses were titrated over time according to disease activity criteria with the aim of achieving clinical remission.

### Nonrheumatoid arthritis patients

Control subjects (*n *= 40) were healthy volunteers recruited from the same geographical area, including 13 men and 27 women with a mean age of 53 years (range 21–80 years), matched to the RA patients' age, sex and CV risk factors. We matched against a database of >1,000 cIMTs recruited in a primary prevention setting. The primary match was for age and gender, after which we matched for number and type of risk factors on a categorical basis. While exact blood pressure or lipid levels were not matched, we were able to match for hypertension and hyperlipidemia, in addition to smoking status in almost all the cases.

### Study procedure

The study was approved by the human research ethics committee at Princess Alexandra Hospital, and all subjects provided written informed consent. RA patients underwent a comprehensive clinical evaluation and measurements by a rheumatologist and a clinical metrologist, including the American College of Rheumatology core set of disease activity criteria, the Health Assessment Questionnaire and calculation of the disease activity score. Demographic variables were ascertained by questionnaire. Disease duration was determined by the length of RA symptoms. Disease activity in RA patients was measured using the disease activity score (DAS 4v), a validated composite score incorporating tender joint counts (out of 53) and swollen joint counts (out of 44), the erythrocyte sedimentation rate, and the patient global assessment of disease activity (100 mm visual analogue scale) [15].

### Cardiovascular risk factor ascertainment

CV risk factors were ascertained among RA patients and controls at presentation. Cigarette smoking was assessed by questionnaire, which included details about past and present smoking habits, the number of cigarettes smoked per day and the smoking duration. Diabetes mellitus was classified as present if diagnosed by a physician or if patients were taking antidiabetic medications.

History of previous angina was classified as present if it had been reported by physician; and history of previous myocardial infarction was classified as present if development of either, first, a typical rise and gradual fall (troponin) or a more rapid rise and fall of biochemical markers of myocardial necrosis (with at least one of ischaemic symptoms, development of pathologic Q waves on ECG (electrocardiogram), or ECG changes indicative of ischaemia (ST-segment elevation or depression)) or, second, either new pathological Q waves on serial ECGs or pathological changes of healed or healing myocardial infarction was reported [16].

History of stroke or transient ischaemic attack was defined by admission to the hospital with computed tomography evidence of ischaemic occlusion or with carotid endarterectomy, or presentation with stroke/transient ischaemic attack symptoms with significant plaque on carotid ultrasound and neurological sequelae, with exclusion of subarachnoid haemorrhage and space-occupying lesions. Family history of CV attack or cerebrovascular attack before age 65 in first-degree relatives was determined by questionnaire.

The body mass index was calculated as weight (kilograms) divided by the square of the height (metres). History of hypercholesterolemia and hypertension were determined by diagnosis and recording as such in medical records by a physician, or use of lipid-lowering drugs or antihypertensive medication. Blood pressure was measured at first presentation.

The 10-year coronary risk was calculated using the Framingham equation. Laboratory data included fasting total cholesterol levels, low-density lipoprotein levels, high-density lipoprotein levels, very-low-density lipoprotein levels, triglyceride levels, glucose levels, C-reactive protein (CRP) levels, the erythrocyte sedimentation rate, haemoglobin levels, anticitrullinated peptide levels, rheumatoid factor levels, antinuclear antibodies, and high-resolution HLA-DRB genotyping.

### Measurement of intima-media thickness and plaque

Analyses were carried out on RA patients within 1–4 weeks after RA diagnosis, and generally before commencement of antirheumatic therapy. Ultrasound images were frozen by ECG triggering (top of R-wave) to minimize variability depending on changes in the cIMT and lumen diameter occurring during the cardiac cycle [7,17]. Offline analysis of the images was performed on a workstation using standard software for automated measurement of the intima-media thickness (IMT) (QLab 4.2.1 with IMT plugin, version 1.1; Philips Ultrasound, Bothell, WA, USA). The algorithm uses a maximum-slope edge-detection technique, beginning in the vessel lumen and detecting the first maximum slope of the change in signal for the near and far walls, and repeating the analysis to identify the media–adventitia interface. The software calculates the mean and standard deviation for the IMT thickness in a region-of-interest box, placed by the observer perpendicular to the vessel walls, with the measurements performed on the R wave of the ECG.

Care was taken not to include any plaque detected in the carotid artery in the IMT measurement. Images were obtained of the anterior, lateral and posterior views from both the right and left carotid arteries; because of extraneous noise and gain dependency in the near field, however, only the measurements of far wall views were recorded. These measurements were averaged to provide a mean IMT. The intra-observer variation and the coefficient of variance of the IMT from this institution are 0.01 ± 0.04 mm (5%). For each subject the average IMT of six frozen images was calculated, and the mean cIMT ((left + right)/2) was taken as a measure of the wall thickness of the distal common carotid artery.

Both extracranial carotid arterial systems were extensively scanned to identify plaque, which was defined as a focal widening relative to adjacent segments, with protrusion into the lumen composed of calcified deposits (hard plaque), noncalcified deposits (soft plaque) or a combination of calcification and noncalcified material. Plaque was considered present if visualized in the full diameter of the vessel; that is, both the proximal and distal parts of the plaque were attached to the typical double-lined intima-media structure and the double lines were also visible on the opposite side of the lumen [18]. Examination and analysis of the cIMT and plaque were performed by the same investigator (SH).

### Statistical analysis

Continuous variables are described as the mean ± standard deviation, and categorical variables presented as the percentage. Log transformations were applied to non-normally distributed data. The chi-squared test was used to analyse categorical variables. cIMT differences between RA patients and controls were tested with a two-sample *t *test. The association of the cIMT with age, continuous CV variables and disease activity variables was evaluated using linear regression analysis.

In the absence of previous cIMT data in early RA, an *a priori *power calculation was not possible concerning the study sample size. Based on an expected IMT of 0.58 ± 0.09 (the mean in middle-aged asymptomatic primary prevention patients in our practice), however, we anticipated that a group of 40 early RA patients and 40 matched control individuals would have a 90% power to permit the detection of the expectation of a 10% greater IMT in RA patients, and a significant difference (*P *< 0.05) in the IMT between RA patients and control subjects. In view of the sample size of 40, the four variables that were significant at *P *≤ 0.01 on univariate linear regression analysis were analysed in a multivariate backwards stepwise linear regression model.

Logistic regression was used to look for correlations between CRP levels <5 and ≥ 5 with traditional CV risk factors. A CRP value of 5 was chosen as a clinically useful discriminator of values above and below the normal reference range.

Mediation analysis was carried out as previously described, where indicated [19]. All analyses used Stata 9/SE statistical software (Stata Corp, College Station, Texas, USA).

## Results

### Clinical features

Forty patients presenting with definite RA within 12 months of symptom onset were studied (Table 1). Three of these patients had previous CV events, including one male with a previous transient ischaemic attack at 52 years of age and two males with angina at age 48 and 64 years, with a myocardial infarction at age 48 in one of those patients. Except for one patient with rheumatoid nodules, no patient had developed extraarticular manifestations at presentation. The most prevalent CV risk factor was history of smoking; patients and controls were matched for age, sex and all traditional CV risk factors, with no statistically significant difference between the two groups (Table 2).

**Table 1:** Demographic details, rheumatoid arthritis characteristics, and laboratory values of 40 rheumatoid arthritis patients at first presentation

Demographic detail	Value
Male:female	13:27
Mean age (years)	53 (22–78)
Male mean age (years)	58 (37–78)
Female mean age (years)	51 (22–72)
Body mass index (kg/m^2^)	28 (17–45)
Rheumatoid arthritis characteristics	
Disease duration (months)	7 (1.0–12)
Tender joint count (out of 53)	19 (14)
Swollen joint count (out of 44)	16 (10)
Patient's assessment of fatigue (visual analogue scale, maximum 100)	38 (28)
Health assessment questionnaire score (maximum disability 24)	4.4 (4.5)
Physician's global assessment of disease activity (maximum 100)	41 (26)
Joint pain (visual analogue scale, maximum 100)	50 (31)
Morning stiffness duration (min)	195 (358)
Rheumatoid factor level	228 (302)
Disease activity score (DAS 4v)	4.39 (1.6)
Rheumatoid factor positive (*n *(%))	30 (75%)
Laboratory values	
Erythrocyte sedimentation rate (mm/hour)	36.5 (24)
C-reactive protein (mg/l, normal <6)	25.5 (23)
Haemoglobin (g/l)	131 (15)
Cholesterol (mmol/l)	5.0 (1.0)
Triglyceride (mmol/l)	1.4 (0.9)
High-density lipoprotein level (mmol/l)	1.5 (0.4)
Total high-density lipoprotein/cholesterol ratio (mmol/l)	3.6 (1.2)
Low-density lipoprotein cholesterol (mmol)	2.8 (0.7)
Antinuclear antibody titre >1:160 (*n *(%))	28 (70%)

Data presented as mean (range) or mean (standard deviation), unless indicated otherwise.

**Table 2:** Established cardiovascular risk factors in 40 patients with early rheumatoid arthritis and in 40 control individuals

Variable	Rheumatoid arthritis patients	Control individuals	*P *value
Cholesterol (mmol/l)^a^	5.0 (1.0)	5.1 (1.0)	0.84
Triglyceride (mmol/l)^a^	1.4 (0.9)	1.4 (0.9)	0.92
low-density lipoprotein cholesterol (mmol/l)^a^	2.8 (0.7)	3.0 (0.9)	0.80
high-density lipoprotein cholesterol (mmol/l)^a^	1.5 (0.4)	1.5 (0.4)	0.87
Body mass index (kg/m^2^)^a^	28 (7.6)	28 (5.7)	0.96
Smoking, ever^b^	26 (65)	24 (60)	0.64
History of hypertension^b^	7 (18)	11 (28)	0.28
History of diabetes mellitus^b^	5 (12)	5 (12)	1
History of hypercholesterolemia^b^	3 (8)	6 (15)	0.29
History of myocardial infarction^b^	1 (2)	0	-
History of angina^b^	2 (5)	0	-
History of stroke and/or transient ischaemic attack^b^	1 (2)	0	-
Family history of cardiovascular disease^b^	6 (15)	10 (25)	0.26
Systolic blood pressure^b^	122 (24)	124 (18)	0.55
Diastolic blood pressure^b^	74 (10)	77 (9.6)	0.19
10-year coronary risk^b^	10(8.7)	9.1(7.4)	0.56

Data presented as ^a^mean (standard deviation) or ^b^*n *(%).

### Carotid intima-media thickness

**Figure 1:**
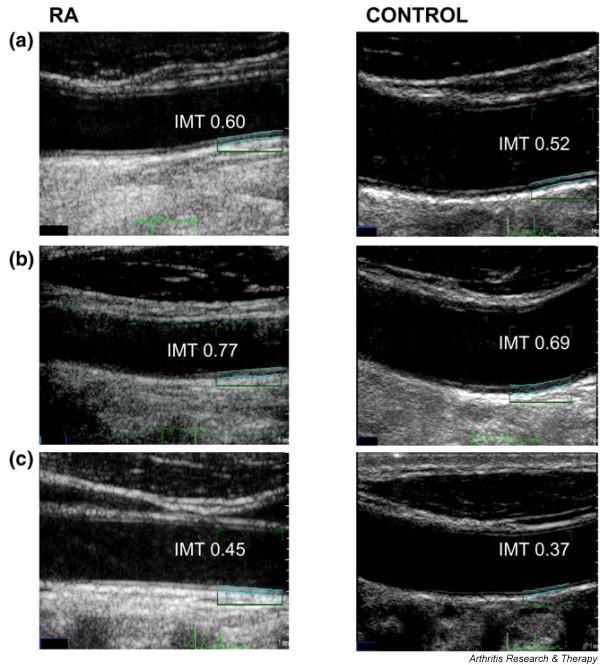
Carotid intima media thickness in rheumatoid arthritis patients compared with matched healthy control individuals. The carotid intima media thickness (IMT) is increased in rheumatoid arthritis (RA) patients compared with healthy control individuals matched for age, sex and cardiovascular risk factors. Representative carotid ultrasound images from three matched RA patient and control individual pairs: **(a) **a 42-year-old male with RA and a 40-year-old control male, **(b) **a 58-year-old male with RA and a 58-year-old control male, and **(c) **a 31-year-old female with RA and a 31-year-old control female. The automated measurement lines over the region of interest are boxed (in blue).

When compared with healthy matched control subjects, RA patients exhibited a greater average cIMT than control subjects (0.64 ± 0.13 mm versus 0.58 ± 0.09 mm, *P *= 0.03) (Figure 1) and an increased prevalence of carotid atherosclerotic plaque (14 (35%) versus 3 patients (7.5%), *P *= 0.01). We repeated the analysis after removing patients with previous CV events and their controls, and the results did not differ (0.63 ± 0.02 mm versus 0.57 ± 0.01 mm, *P *= 0.03). These data indicate that patients presenting with early RA have increased atherosclerotic burden as evidenced by increased cIMT and carotid arterial plaque, compared with matched control subjects.

### Determinants of carotid intima-media thickness

**Figure 2:**
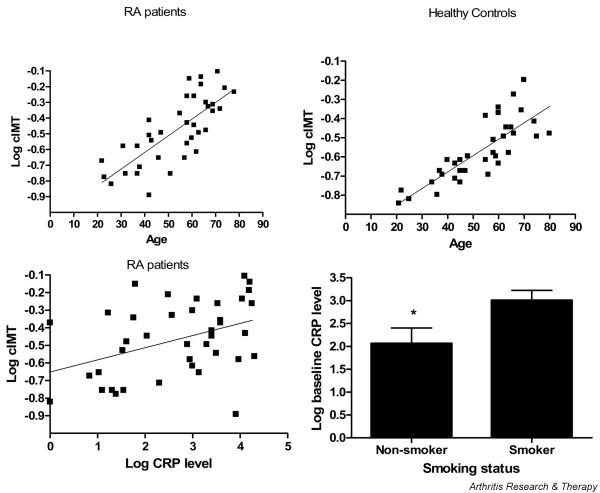
Coassociation of carotid intima media thickness, age and rheumatoid arthritis inflammatory activity. The carotid intima media thickness (cIMT) was measured in 37 rheumatoid arthritis (RA) patients with disease duration <12 months and in 37 control subjects matched for age, sex and cardiovascular risk factors. The cIMT was plotted against (top left) age at RA diagnosis, (top right) age of controls, or (bottom left) the C-reactive protein (CRP) level at first presentation with RA. (Bottom right) CRP at first presentation plotted against history of smoking. Data presented as mean ± standard deviation. * p < 0.05

Univariate and multivariate analyses were carried out to determine CV risk factors or inflammatory factors that might contribute to atherosclerosis in early RA. A positive association was shown between the cIMT and the CRP level, the disease activity score (DAS 4v), age, male sex, the pack-year history of smoking, history of smoking and systolic blood pressure at the time of presentation with RA (Table 3 and Figure 2). The slope of the regression line with age was steeper in RA patients (slope = 0.011, *R*^2 ^= 0.58) than in control individuals (slope = 0.009, *R*^2 ^= 0.66), suggesting a more rapid rate of thickening of cIMT in RA patients than controls with increasing age (Figure 2). RA patients with previous CV events and their matched control subjects were removed for this analysis. After addition of RA as an interactive term to the regression analysis, the presence of RA did not affect the relationship between age and cIMT. The role of inflammatory activity became clearer upon multivariate analysis, which revealed a strong association between the cIMT, age and CRP level at presentation (Table 3, adjusted *R*^2 ^= 0.64).

**Table 3:** Linear regression analysis of carotid intima-media thickness, rheumatoid arthritis features and cardiovascular risk factors in recent-onset rheumatoid arthritis patients

Variable	*R*^2 ^value	Standardized β coefficient	Standard error	*t *value	*P *value	Confidence interval
Univariate model						
Number of swollen joints	0.12	0.391	0.041	2.25	0.032	0.008–0.174
Disease activity score (DAS4v)^a^	0.16	0.431	0.079	2.74	0.01	0.056–0.377
C-reactive protein level (mg/l)^a^	0.14	0.407	0.025	2.71	0.01	0.018–0.121
Rheumatoid factor presence^b^			0.047		0.54	0.072–0.117
Age at onset of rheumatoid arthritis^a^	0.56	0.759	0.001	7.19	<0.001	0.008–0.014
Male sex^b^			0.066		0.018	-0.298 to -0.030
Pack-year history	0.10	0.320	0.001	2.09	0.044	0.000–0.005
History of smoking, ever^b^			0.062		0.002	-0.329 to -0.079
Systolic blood pressure^a^	0.27	0.540	0.184	3.63	<0.001	0.292–1.040
Cholesterol level	0.01	0.146	0.197	0.82	0.417	-0.564 to -0.239
Multivariate model						
Age at onset of rheumatoid arthritis	0.64		0.002	6.11	<0.001	0.007–0.012
C-reactive protein level (mg/l)			0.001	2.18	0.038	0.000–0.004

Adjusted *R*^2 ^of the multivariate linear regression model = 0.69. ^a^Variables entered into the multivariate model. Parameters determined by univariate linear regression analysis or *t *test^b^.

### Determinants of C-reactive protein

We hypothesized that CRP and other markers of inflammation were major contributors to the cIMT in RA, and this was indeed the case. To further understand contributors to the cIMT in early RA, we determined the relationship between the CRP level at presentation and traditional CV risk factors. The CRP level at disease onset was related to age (*P *= 0.016; Figure 2), and with each yearly increase in age there was a corresponding 5% increase in probability of CRP > 5 (*P *= 0.035). The low-density lipoprotein cholesterol level and triglycerides showed a positive linear relationship to CRP, and the high-density lipoprotein cholesterol level showed a negative linear relationship to CRP level at disease onset (*P *< 0.05). Presence of CRP > 5 was significantly associated with a history of smoking (*P *= 0.015). Those who had ever smoked were at greater risk of CRP > 5, with an odds ratio of 5.6 (*P *= 0.019). History of smoking was the only variable to maintain a significant association with CRP level at presentation in a multivariate model (*P *= 0.02). Further analysis demonstrates no mediation of CRP on the relationship of cIMT with traditional CV risk factors, except for body mass index.

## Discussion

In the present study, we sought evidence of subclinical atherosclerosis by carotid ultrasound in patients presenting with recent-onset RA. Using this technique, the cIMT or carotid plaque are markers of atherosclerotic burden, and correlate with CV outcome [4-6]. Patients with established RA have been shown to exhibit significantly greater cIMT or plaque than matched control subjects [8-14]. Our data are the first to show an increased cIMT and increased plaque, and thus show accelerated atherosclerosis in patients at first presentation with symptoms of RA. While our study was too small to effectively model factors associated with cIMT in early RA, age and baseline CRP emerged from our analysis as the key determinants of cIMT in early RA, which deserve further testing in future studies.

Age and the cIMT were closely associated in both our patient and matched healthy control populations. This reflects previous studies of cIMT in established RA patients and in the general population [9,13,20]. Although the control group showed a similar association between IMT and age, the RA patients' cIMT was significantly higher than that of the controls. This indicates that while age is a strong risk factor for increased cIMT, it is not enough to explain the increased incidence of subclinical atherosclerosis in RA patients. In addition, the baseline CRP level showed a significant relationship with age, suggesting that patients who develop RA later in life tend to have higher levels of inflammation at first presentation, putting them at higher risk of CV and atherosclerotic disease.

In support, previous studies identified inflammatory activity to be important for the development of CV disease in RA, even after adjustment for traditional CV risk factors [21-24]. The consistent association of cIMT with inflammatory markers supports the evidence that CV mortality in RA patients is associated with inflammation. Previous studies have consistently shown that excess CV mortality in RA is associated with baseline or subsequent evidence of high levels of inflammation, including elevated CRP, elevated erythrocyte sedimentation rate and elevated swollen joint counts [5,25-27]. Several studies also demonstrate the association of older age at RA onset, smoking, hypertension and male gender on CV mortality [5,25-27]. We further show here that CRP is a major risk factor for atherosclerosis in patients first presenting with RA. The current data identify the same set of variables – age, smoking, blood pressure and indices of disease activity – to be associated with increased cIMT in early RA as are associated with CV mortality. Moreover, inflammatory and traditional CV risk factors are correlated rather than acting independently on cIMT.

The cIMT assesses the atherosclerotic burden and provides information that is independent and incremental to that provided by standard risk factors. Noninvasive tools such as ultrasound measurement of the cIMT may be useful to better understand risk levels in primary prevention [28]. Age-specific cIMT nomograms should be helpful in determining age-appropriate cIMT values [20], and may be used to identify subclinical atherosclerosis in RA patients. This information could be used in decision-making regarding preventive measures including statin therapy [29], although further studies are needed to define whether this strategy is effective. In addition to monitoring CV risk factors and inflammatory activity, this technique might be useful to monitor progression of atherosclerosis over time [21].

Smoking is clearly a risk factor for RA development, smoking exacerbates RA severity, and smoking is a risk factor for the IMT and CV outcome in RA and non-RA patients [5,25,30-35]. Moreover, smoking promotes the development of anticitrullinated peptide autoantibodies in individuals with RA susceptibility HLA-DRB1 alleles, occurring potentially years before the development of clinical RA [36,37]. Our study demonstrates significant associations between the cIMT, smoking and CRP in early RA patients, supporting the concept that smoking is associated with more severe inflammation at disease presentation. Indeed, considering that 65% of participants had a history of smoking, it is difficult to separate the influence on the cIMT of CRP levels alone from CRP levels promoted by the oxidative environment of smoking. The data therefore strongly suggest inflammation that precedes the onset of joint symptoms in RA promotes the development of atherosclerotic disease well before the first manifestations of joint disease, and that the pathogenesis of atherosclerosis is linked to that of autoimmune joint disease [38-40]. This conclusion is consistent with previous studies demonstrating either early CV mortality after onset of inflammatory polyarthritis or a lack of association of disease duration with CV mortality, suggesting that the increased risk of CV death was already present at RA diagnosis [5,25]. Our data strongly emphasize the importance of controlling CV risk factors, such as hypertension, smoking and inflammatory activity early in the RA disease course, and emphasize the critical influence of smoking on inflammation in RA.

## Conclusion

Early RA patients have increased atherosclerotic burden compared with matched control individuals, predicted by age and baseline CRP. Since inflammation has been shown to predate the onset of clinical RA, the accelerated atherogenic process related to inflammation may precede RA symptom onset.

## Abbreviations

cIMT = carotid intima-media thickness; CRP = C-reactive protein; CV = cardiovascular; ECG = electrocardiogram; IMT = intima-media thickness; RA = rheumatoid arthritis.

## Competing interests

The authors declare that they have no competing interests.

## Authors' contributions

SH, BH, THM and RT were all involved in the conception, design, acquisition, analysis and interpretation of the data. SH, THM and RT drafted and revised the manuscript. All authors read and approved the final manuscript.
